# A multi-centre investigation of macrovascular and non-ocular microvascular complications in children and adolescents with diabetes mellitus in southern Ghana

**DOI:** 10.4314/gmj.v57i2.2

**Published:** 2023-06

**Authors:** Josephine Akpalu, Vera A. Essuman, Winfried M. Amoaku, Benjamin Abaidoo, Akye Essuman, Charles Hayfron-Benjamin, Nana A. Barnes, Naa N. Tagoe, George Asare, Thomas A. Ndanu, Benedicta Appiah-Thompson, Imelda D-B. Ofori-Adjei, Adziri H. Sackey

**Affiliations:** 1 Department of Medicine and Therapeutics, University of Ghana Medical School, University of Ghana, Accra, Ghana; 2 Ophthalmology Unit, Department of Surgery, University of Ghana Medical School, University of Ghana, Accra, Ghana; 3 Academic Ophthalmology, MHCN, University of Nottingham, ‘B’ Floor, Eye & ENT Centre, Nottingham University Hospital, QMC Nottingham, UK; 4 Department of Internal Medicine, University of Health and Allied Sciences, Ho, Ghana; 5 Department of Physiology, University of Ghana Medical School, University of Ghana, Accra, Ghana; 6 Department of Anaesthesia, Korle Bu Teaching Hospital, Korle Bu, Accra, Ghana; 7 Santa Rosa Community Health, Vista Clinic 3569 Round Barn Circle, Santa Rosa, USA; 8 Eye Department, Korle Bu Teaching Hospital, Accra, Ghana; 9 Chemical Pathology Unit, Department of Medical Laboratory Sciences, University of Ghana School of Basic and Allied Health Sciences, College of Health Sciences, Accra, Ghana; 10 Department of Preventive & Community Dentistry, University of Ghana Dental School, University of Ghana, Accra, Ghana; 11 Eye Department, Cape-Coast Teaching Hospital, Cape Coast, Ghana; 12 Department of Child Health, University of Ghana Medical School, University of Ghana, Accra, Ghana

**Keywords:** Diabetes complications, children, adolescents, Ghana

## Abstract

**Objectives:**

To investigate the prevalence of macrovascular and non-ocular microvascular complications and the associated factors among children and adolescents with diabetes mellitus in selected hospitals in southern Ghana.

**Design:**

A cross-sectional study.

**Setting:**

The out-patient clinics of the Departments of Child Health, Medicine and Therapeutics, Family Medicine, Ophthalmology, and the National Diabetes Management and Research Centre, all at the Korle Bu Teaching Hospital, Accra, as well as from Cape-Coast Teaching Hospital in the Central Region of Ghana.

**Participants:**

Fifty-eight children and adolescents aged 4-19 years who had been diagnosed with diabetes mellitus.

**Main outcome measures:**

Macrovascular (peripheral artery disease and coronary heart disease) and non-ocular microvascular complications (neuropathy and nephropathy).

**Results:**

Data from 58 children and adolescents with diabetes were analysed. The mean age of participants was 14.6±2.6 years, and a female preponderance was observed (45, 77.6%). The prevalence of macrovascular and non-ocular microvascular complications was 27.6% and 8.6%, respectively. Long duration of diabetes diagnosis (p=0.044) and low triglycerides (p=0.009) were associated with microvascular complications, while high triglycerides (p=0.032), lower HDL cholesterol (p=0.046), and abnormal body mass index (p=0.020) were associated with macrovascular complications.

**Conclusions:**

Macrovascular and non-ocular microvascular complications are common among children and adolescents with diabetes in southern Ghana and are associated with a long duration of diabetes diagnosis, abnormal body mass index, low HDL cholesterol, and triglyceride levels. Therefore, the early institution of regular screening for diabetes-related complications to allow early detection and appropriate management is recommended.

**Funding:**

University of Ghana Research Fund

## Introduction

The worldwide prevalence and incidence of diabetes mellitus (DM), be it type 1 (T1) or 2 (T2), among children and adolescents are on the increase.^1^ The development of DM at a young age is associated with an increased risk of long-term microvascular and macrovascular complications.^2^ Whereas microvascular complications (diabetic retinopathy, diabetic nephropathy, and diabetic neuropathy) are specific to DM, macrovascular complications comprising atherosclerotic cardiovascular diseases (ASCVD) are not. Nonetheless, the risk of macrovascular complications is higher among patients with DM than those without DM.^3^

The prevalence of DM-related complications among young adults diagnosed with DM in childhood and adolescence was high, with 32% and 72% of T1DM and T2DM patients reported to have at least one long-term complication.^2^ A high prevalence of chronic DM-related complications has also been reported in young individuals from sub-Saharan Africa (SSA).^4^,^5^

In individuals with the onset of DM at a young age, these complications often develop after prolonged exposure to risk factors, with the acceleration of complications occurring during puberty.^6^ This acceleration is reportedly due to hormonal changes at puberty and the sub-optimal glycaemic control that occurs during this period.^7^ Risk factors for microvascular and macrovascular complications include young age at diagnosis, longer duration of diagnosis, hyperglycaemia, hypoglycaemia, obesity, hypertension, and low socio-economic status.^8^,^9^ Diabetes in children and adolescents is associated with an increase in all-cause and cardiovascular mortality, compared with those without DM.^10^ Optimal diabetes care in children and adolescents is essential to prevent or reduce the morbidity and mortality associated with vascular complications in their adult years.^11^ A decline in the incidence of diabetes-related complications has been reported elsewhere.^12^ Although the contributory factors for this reduction are not fully elucidated, it may be due to significant improvements in DM care, including the implementation of regular screening for complications and their risk factors and modern diabetes technology.^12^ Other measures include educating patients and caregivers and adopting multi-disciplinary management.^3^ Limited data from SSA, where diabetes care is often not optimal, indicates that the prevalence of chronic vascular complications among the youth with DM remains high.^11^,^13^ In Ghana, the burden of chronic complications of diabetes among children and adolescents has not been investigated.^14^ This study, therefore, aimed at determining the prevalence of macrovascular and non-ocular microvascular complications and the associated factors among children and adolescents with DM in selected hospitals in southern Ghana. The findings are expected to bridge this knowledge gap and provide information clinicians, and policymakers can use to improve diabetes care in children and adolescents.

## Methods

### Study design and setting

This cross-sectional study investigated DM-related complications in children and adolescents in southern Ghana. Study participants were recruited at the out-patient clinics of the Departments of Child Health, Medicine and Therapeutics, Family Medicine, Ophthalmology, and the National Diabetes Management and Research Centre, all at the Korle Bu Teaching Hospital, Accra, as well as from Cape-Coast Teaching Hospital in the Central Region of Ghana from August 2017 to September 2019. Our earlier manuscript published elsewhere reports further study design and protocol details.^14^

### Study participants

Study participants were children and adolescents aged 4-19 years who had previously been diagnosed with DM based on fasting plasma glucose of ≥ 7mmol/L or random plasma glucose of ≥ 11.1 mmol/L (repeated or with classic symptoms of hyperglycaemia).^15^ Study participants were consecutively recruited after written informed consent or assent was obtained from each guardian or participant, respectively. Children and adolescents aged 4-19 years who did not assent to or whose parents/ guardians did not give consent for inclusion and children under four years or older than 19 were excluded from the study.

### Data collection

A data collection form was used to gather participants' demographic, clinical and laboratory information. The data included age, sex, age at diagnosis, duration of diagnosis, type of DM, type of treatment (insulin, oral glucose-lowering medications or both), and family history of DM.

A full physical examination and investigations were performed for all participants to determine the presence of microvascular and macrovascular complications and other co-morbidities associated with DM. The blood pressure (lying and standing) was documented. Hypertension was defined as systolic or diastolic blood pressure greater or equal to the 95^th^ centile for age, sex and height. Participants' height in metres(m) and weight in kilogrammes(kg) were measured, and their body mass index (BMI) (kg/m^2^) was determined. Age and sex-standardized BMI z-scores were computed and defined using the World Health Organization (WHO) growth standards for BMI in children and adolescents.^16^ Diabetes was further characterized as T1DM by the presence of GAD 65 antibodies, islet cell antibodies, and low levels of C-peptide (stimulated C peptide values of <0.7 pmol/mL); or T2DM, indicated by a negative test for T1DM-specific antibodies in association with elevated fasting insulin or C-peptide (stimulated C-peptide assay of ≥ 1.9 pmol/mL), or the presence of acanthosis nigricans or obesity. Blood C-peptide levels were determined by competitive enzyme immunoassay methods using ELISA kits (NovaTec Immundiagnostica GmbH, Germany). Islet Cell Antibodies were assayed using Islet Cell Ab (ICA) Enzyme Immuno Assay Kits (Gateways Medical dba BMS-USA). GAD65 autoantibodies were assayed using another ELISA kit (Medizym anti-GAD Testkit 96 tests, MEDIPAN GMBH, Germany).

### Microvascular complications

Clinical assessment for diabetic peripheral neuropathy included a history of neuropathic symptoms, physical examination as recommended by the American Diabetes Association, and assessment of vibration perception threshold (VPT).^15^ Participants' feet were examined for deformities, ulcers, calluses, pinprick sensation, and vibration sensation using a 128-Hz tuning fork and the 10-g monofilament testing was done. According to the manufacturer's guidelines, VPT was assessed using the hand-held neurothesiometer (Horwell Neurothesiometer, Scientific Laboratory Supplies Ltd, Nottingham, UK). VPT was assessed at the metatarsophalangeal joint of both feet in a two-step manner, starting from 0 V with increasing stimulation and then starting from 50 V with decreasing stimulation. Patients with a VPT greater than 15V, with or without clinical features of peripheral neuropathy, were classified as having diabetic peripheral neuropathy. Nephropathy was diagnosed using the estimated Glomerular Filtration Rate (eGFR) of < 90ml/min/1.73m^2^. Blood urea (Urease UV method), electrolytes (the ion selective electrodes and enzymatic method for bicarbonates), and creatinine (Jaffe IDMS Traceable method) were assayed with the AU480 Beckman Coulter, USA.

### Macrovascular complications

Diagnosis of peripheral arterial disease was based on examining peripheral pulses and measuring the Ankle Brachial Pressure Index (ABI).^17^ An 8 MHz Doppler probe (Huntleigh, UK) was used to determine flow reappearance during slow deflation of a blood pressure cuff. Systolic BP was measured twice in the brachial artery of each upper arm and twice in the posterior tibial or dorsalis pedis arteries at each ankle. ABI was calculated using the higher brachial systolic BP as the denominator and the lower ankle systolic BP as the numerator. The lower left and right ABI measurements were used for the analysis. An ABI ≤ 0.90 was indicative of peripheral arterial disease.^17^

Coronary Artery Disease was assessed using a 12-lead electrocardiogram (Nova PC -based ECG system) and analysed using the Minnesota Code.

### Other laboratory investigations

Glycated haemoglobin (HbA1c) was measured using the Tri-Stat Boronate Affinity System (Trinity Biotech, Ireland). Patients with HbA1c <7.0% and ≥7.0% were said to have good or poor glycemic control, respectively.^15^ Lipid profile was assayed by determining the total cholesterol (Chol oxidase, esterase), triglycerides (Enzymatic endpoint), HDL cholesterol (Direct measure polymer-polyanion) and LDL cholesterol by enzymatic methods using an automated chemistry analyzer (AU480 Beckman Coulter/USA). Total cholesterol < 4.4mmol/L, LDL cholesterol < 2.6mmol/L, HDL cholesterol > 1.0 and triglycerides < 1.0 were considered normal.

### Data Analysis

Data were analysed using the Statistical Package for Social Sciences (SPSS Version 25.0). Normality and similarity of data distribution were assessed with the Kolmogorov-Smirnov test. Results of the analysis were expressed as mean±standard deviation (SD) for continuous variables normally distributed, median (Inter-Quartile-Range) for non-normally distributed variables and counts and percentages for categorical variables. The prevalence of macrovascular and non-ocular microvascular complications in children and adolescents with diabetes was computed and presented as percentages. The chi-square or Fisher's exact tests assessed associations between socio-demographic and clinical factors and microvascular and macrovascular complications. The association between biochemical variables and microvascular and macrovascular complications was computed using the Mann-Whitney-U and ANOVA tests. P-values less than 0.05 were considered statistically significant.

### Ethical consideration

Approval was obtained from the Ethical and Protocol Review Committee of the College of Health Sciences, University of Ghana (Ref: MS-AA/C.2/Vol.18) and Cape-Coast Teaching Hospital (Ref: CCTH/MD-G/14-0154). This study also followed the principles of the Helsinki Declaration of 2014.

## Results

### Demographic and clinical characteristics of children and adolescents with diabetes

Fifty-eight (58) children and adolescents with DM were recruited. The mean age of participants was 14.6±2.6 years, with a female preponderance (45, 77.6%). Most (36, 62.1 %) participants were at the basic level of education.

The mean age at diagnosis of DM was 10.8±3.1 years, with the median duration of DM being 3.0 (1.0-6.3) years. The minimum and maximum duration of DM was 4.0 months and 13.0 years, respectively. Fifty-four (93.1%) participants were classified as having T1DM. Thirty-eight (65.5%) participants had a family history of diabetes. Most participants (34, 89.5%) had T1DM (Table 1).

**Table 1:** Demographic and clinical characteristics of Ghanaian children and adolescents with DM

Characteristics	Number	Percentage (%)	Mean±standard deviation	Median (interquartile range)
**Age:**			14.6±2.6	15.0(13.0-16.0)

**<12**	6	10.3		

**12-19**	52	89.7		

**Sex:**				

**Male**	13	22.4		

**Female**	45	77.6		

**Educational level:**				

**Basic**	36	62.1		

**SHS**	21	36.2		

**Tertiary**	1	1.7		

**Duration of DM (yrs):**			4.0±3.4	3.0(1.0-6.3)

**<5**	38	65.5		

≥**5**	20	34.5		

**Age at diagnosis (yrs)**			10.8±3.1	11.0(9.0-13.3)

**BMI**			20.7±4.9	20.2(17.6-22.3)

**BMI category: (z-score)**				
**Severe thinness (< -3)**	3	5.2		
**Thinness (-3 to -2)**	4	6.9		
**Normal (-2 to 1)**	43	74.1		
**Overweight (1 to 2)**	6	10.3		
**Obese (> 2)**	2	3.4		

**Type of diabetes:**				

**Type 1**	54	93.1		

**Type 2**	4	6.9		

**Type of treatment:**				

**Insulin only**	53	91.4		

**Oral glucose lowering drugs only**	3	5.2		

**Combination**	2	3.4		

**Type of insulin:**				

**Premixed insulin**	55	94.8		

**Type of oral medication:**				

**Metformin**	5	8.6		

**Family hx of DM**	38	65.5		
**Mother only**	20	34.5		
**Father only**	10	17.2		
**Siblings**	3	5.2		
Others *	5	8.6		

**HbA1c**			10.1±2.9	9.9(8.0-12.4)

**High blood pressure**	9	15.5		

**Blood pressure:**				

**Lying systolic**			113.6±12.5	110.0(106.0-121.3)

**Lying diastolic**			67.3±9.0	68.5(62.0-71.3)

**Standing systolic**			111.4±10.7	111.0(104.0-119.3)

**Standing diastolic**			71.3±9.1	70.0(66.0-75.3)

**Pulse rate**			84.3±11.4	84.5(77.0-90.0)

DM = Diabetes Mellitus, Basic education (from primary 1 to Junior High School), SHS = Senior High School, BMI = Body Mass Index, hx = history, HbA1c = Glycated haemoglobin.

* Includes: maternal grandfather = 2(3.4%), maternal uncles = 2(3.4%); maternal aunt = 1(1.7%).

Five (8.6%) participants had a goitre, and acanthosis nigricans was present in 4 (6.9%) participants on physical examination. Most participants (47, 74.1%) had normal BMI. High blood pressure was present in 9 (15.5%) participants. The main pharmacological treatment for diabetes was premixed insulin (55, 94.8%). The mean HbA1c was 10.1±2.9 %, and the median HbA1c was 9.9 (8.012.4) % (Table 1).

### Prevalence of non-ocular microvascular and macrovascular complications in children and adolescents with diabetes

A total of 5 (8.6%) participants had non-ocular microvascular complications, and 16 (27.6 %) had macrovascular complications. Nephropathy was diagnosed in 4 (6.9%) participants, neuropathy in one (1.7%), and coronary artery disease in 16 (27.6%). No peripheral artery disease was detected.

### Association between socio-demographic factors, clinical factors, and DM-related complications

Abnormal BMI (p = 0.020) and long duration of DM diagnosis (p=0.044) were associated with macrovascular and non-ocular microvascular complications, respectively. No statistically significant association was found between other clinical or socio-demographic factors and DM-related vascular complications (p> 0.05). (Table 2).

**Table 2:** Prevalence of macrovascular and non-ocular microvascular complications in Ghanaian children and adolescents with DM

Factors	Complications					

	Non-ocular Microvascular			Macrovascular		

	Yes	No	P-value	Yes	No	P-value
**Age(yrs):**						

**<12**	1(16.7)	5(83.3)	0.433	1(16.7)	5(83.3)	1.000

**12-19**	4(7.7)	48(92.3)		15(28.8)	37(71.2)	

**Sex:**						

**Male**	2(15.4)	11(84.6)	0.311	5(38.5)	8(61.5)	0.482

**Female**	3(6.7)	42(93.3)		11(24.4)	34(75.6)	

**Education:**			0.942			0.418

**Basic**	3(8.3)	33(91.7)		12(33.3)	24(66.7)	

**SHS**	2(9.5)	19(90.5)		4(19.0)	17(81.0)	

**Tertiary**	-	1(100)		-	1(100)	

**Duration of DM (yrs):**						

**<5**	1(2.6)	37(97.4)	0.044*	12(31.6)	26(68.4)	0.538

≥**5**	4(20.0)	16(80.0)		4(20.0)	16(80.0)	

**Type of diabetes:**						

**Type 1**	4(7.4)	50(92.6)	0.310	14(25.9)	40(74.1)	0.303

**Type 2**	1(25.0)	3(75.0)		2(50.0)	2(50.0)	

**Family hx of DM**	4(10.5)	34(89.5)	0.650	13(34.2)	25(65.8)	0.215

**High blood pressure**	1(11.1)	8(88.9)	0.584	2(22.2)	7(77.8)	0.523

BMI category:(z score)			0.473			0.020*
Severe thinness (< -3)	1(33.3)	2(66.7)		3(100.0)	-	
Thinness (-3 to -2)	-	4(100)		1(25.0)	3(75.0)	
Normal (-2 to 1)	3(7.0)	40(93.0)		8(18.6)	35(81.4)	
Overweight (1 to 2)	1(16.7)	5(83.3)		3(50.0)	3(50.0)	
Obese (> 2)		2(100)		1(50.0)	1(50.0)	

**Goitre**	1(20.0)	4(80.0)	0.374	2(40.0)	3(60.0)	0.609

**Acanthosis nigricans**	1(25.0)	3(75.0)	0.310	3(75.0)	1(25.0)	0.060

DM= Diabetes mellitus; hx = history; yrs = years.

* Statistically significant.

Twenty-one (36.2 %) participants had a history of diabetic ketoacidosis (DKA); one participant (1.7%) reported one episode of DKA, and 20 (34.5 %) had two or more episodes of DKA over two years preceding their recruitment. Over the same period, 26 (44.8 %) participants had experienced hypoglycaemia; 7 (12.1 %) participants reported one episode of hypoglycaemia, 4 (6.9%) reported two episodes, and 15 (25.9%) participants reported three or more episodes (Table 3). There was no association between acute and chronic (non-ocular microvascular and macrovascular) DM complications (p> 0.05).

**Table 3:** Association between acute and chronic diabetes complications in Ghanaian children and adolescents

Findings	Complications						
	Microvascular			Macrovascular			Total
	Yes	No	P-value	Yes	No	P-value	N(%)
**DKA**	1(4.8)	20(95.2)	0.644	4(19.0)	17(81.0)	0.365	21(36.2)
DKA hx*							
**1 episode**	-	1(100.0)	1.000	1(100.)	-	0.276	1(1.7)
≥ **2 episodes**	1(5.0)	19(95.0)	0.650	3(15.0)	17(85.0)	0.215	20(34.5)
**Hypoglycaemia**	3(13.0)	23(87.0)	0.648	7(26.9)	19(73.1)	0.578	26(44.8)
Hypoglycemia hx*							
**1 episode**	2(28.6)	5(71.4)	0.106	-	7(100)	0.173	7(12.1)
**2 episodes**	1(25.0)	3(75.0)	0.310	2(50.0)	2(50.0)	0.303	4(6.9)
≥**3 episodes**	-	15(100)	0.313	5(33.3)	10(66.7)	0.738	15(25.9)

### Association between biochemical variables and non-ocular microvascular and macrovascular complications

The median (interquartile range) values for the biochemical variables were: HbA1c 9.9 (8.0-12.4) %, total cholesterol 3.7 (2.9-4.3) mmol/L, triglyceride 1.2 (1.0-1.8) mmol/L, HDL cholesterol 0.4 (0.2-0.6) mmol/L, and LDL cholesterol 3.2 (2.2-3.8) mmol/L. Low triglyceride level was associated with non-ocular microvascular (p=0.009), whiles lower HDL cholesterol (p = 0.046) and high triglycerides (p=0.032) were associated with macrovascular complications (Table 4).

**Table 4:** Association between biochemical findings and DM-related vascular complications in children and adolescents

Biochemical findings	Complications						Total
	Microvascular			Macrovascular			
	Yes	No	P-value	Yes	No	P-value	
**HbA1c (%):**			0.523			0.678	
***Good control (<7%)***	-	8(100)		3(37.5)	5(62.5)		
***Poor control (≥7%)***	4(8.5)	43(91.5)		13(27.7)	34(72.3)		
***Median (IQR)***	11.8(10.2-12.3)	9.6(7.7-12.4)	0.285	9.8(8.2-12.4)	10.3(7.7-12.4)	0.810	9.9(8.0-12.4)
**Total cholesterol (mmol/L)**			0.619			0.702	
***Good(<4.4)***	4(9.1)	40(90.9)		13(29.5)	31(70.5)		
***Borderline(4.4-5.1)***	1(14.3)	6(85.7)		2(28.6)	5(71.4)		
***High(≥5.2)***	-	7(100)		1(14.3)	6(85.7)		
***Median (IQR)***	4.0(3.3-4.6)	3.6(2.9-4.4)	0.551	3.6(2.8-4.3)	3.8(2.9-4.5)	0.670	3.7(2.9-4.3)
**Triglyceride (mmol/L):**			0.008*			0.032*	
***Good(<1.0)***	4(33.3)	8(66.7)		5(41.7)	7(58.3)		
***Borderline(1.0- <1.5)***	1(3.7)	26(96.3)		3(11.1)	24(88.9)		
***High (≥1.5)***	-	19(100)		8(42.1)	11(57.9)		
***Median (IQR)***	0.7(0.5-1.0)	1.3(1.0-1.8)	0.009*	1.5(0.8-2.2)	1.2(1.0-1.5)	0.391	1.2(1.0-1.8)
**HDLc (mmol/L):**			0.757			0.724	
***High(≥1.0)***	-	1(100)		-	1(100)		
***Low(<1.0)***	5(8.8)	52(91.2)		16(28.1)	41(71.9)		
***Median (IQR)***	0.3(0.1-0.5)	0.4(0.2-0.6)	0.283	0.3(0.1-0.5)	0.4(0.3-0.6)	0.046*	0.4(0.2-0.6)
**LDLc (mmol/L):**			0.467			0.510	
***Good(<2.6)***	1(5.6)	17(94.4)		6(33.3)	12(66.7)		
***High(≥2.6)***	4(10.8)	33(89.2)		8(21.6)	29(78.4)		
***Median (IQR)***	3.3(2.8-3.9)	3.1(2.1-3.8)	0.454	3.2(2.2-3.8)	3.2(2.2-3.8)	0.749	3.2(2.2-3.8)

## Discussion

This study investigated the prevalence of non-ocular microvascular and macrovascular DM complications and associated socio-demographic and clinical factors among children and adolescents, and to the best of our knowledge, is the first in Ghana and one of the few in the African sub-region.^14^

The majority of the participants had T1DM, with the mean age of diagnosis and the mean duration of diagnosis comparable to that previously reported in the subregion.^11^ Although the overall incidence of T1DM among children and adolescents is rising globally, a decline has been noted in our setting. This may be due to the high DKA-related mortality in this population at first presentation, even before the diagnosis is made.^18^–^20^ Poor facilities and misdiagnosis are among contributory factors, and prompt recognition and appropriate management are essential to avoid such adverse outcomes.^20^

The global rise in incidence rates has been attributed to an increasing role of environmental factors such as maternal weight gain in pregnancy, rapid linear growth, and weight gain during early childhood which may promote pancreatic islet autoimmunity and the progression to T1DM.^21^ Significantly, a family history of diabetes was reported in many of our participants, although the majority had T1DM. Generally, a family history of DM is associated with an increased risk of T2DM,^18^ although familial aggregates of T1DM have been reported. 22 An increasing prevalence of T2DM among children and adolescents is mainly driven by the rising trend in obesity.^23^ A recent analysis reported the combined prevalence of obesity and overweight among Ghanaian children and adolescents to be high.^24^ With the rising trend of obesity in this population, a concerted effort is required to stem this tide to reduce the burden of DM in young individuals.

Risk factors for DM-related complications include hyperglycaemia, hypertension, dyslipidaemia, obesity, insulin resistance, hypoglycaemia, smoking, age, male sex, long duration of diabetes, young age at diagnosis and low socio-economic status.^3^,^8^,^9^

The poor glycaemic control in our study cohort is similar to that reported from other SSA studies, which have reported mean HbA1c greater than 10%.^4^,^5^,^11^ Factors that account for the poor glycaemic control include the onset of puberty and its attendant hormonal changes (elevated levels of growth hormone, catecholamines, cortisol), reduced parental guidance, oppositional behaviour, poor dietary habits, poor self-monitoring of blood glucose and issues related to insulin use.^4^,^25^ Hyperglycaemia is a well-recognised risk factor for diabetes-related complications. Intermittent hyperglycaemia is associated with additional risk.^26^ It is recommended that HbA1c and time-in-range should be employed in assessing glycaemic control to guide therapeutic options and goals.^27^

The prevalence of nephropathy in this study was lower than that documented among similar study populations from Congo (21%) and Tanzania (29.3%).^4^,^11^ The differences in prevalence may be due to differences in the age distribution, duration of DM diagnosis, and methodology of the different studies. A similar prevalence of neuropathy was found among DM patients in Congo; however, higher prevalence rates have been reported in other studies.^3^,^11^ Variations in the definition of neuropathy and characteristics of the study population may account for the observed differences.

Long duration of diabetes diagnosis, a well-established risk factor for DM-related complications, was significantly associated with microvascular complications in this study.^8^ Lower triglyceride level was significantly associated with microvascular complications in our study, contrary to reports from other studies in which hypertriglyceridaemia has been identified as an emerging risk factor for microvascular complications.^28^,^29^ This disparity may be due to differences in study settings and the other studies having been conducted among adult DM populations.

Individuals diagnosed with diabetes at a younger age have a much higher risk of developing ASCVD than those diagnosed later in life.^30^ ASCVD is a major cause of mortality among adults with onset of DM at a young age, particularly for T2DM.^2^,^31^ The prevalence of coronary artery disease in the present study is much higher than that reported in other studies, and this may be due to the differences in the methodology for diagnosing macrovascular disease.^2^,^32^ Measurement of arterial stiffness, used in other studies, is one of the recommended non-invasive surrogate markers for atherosclerosis and may more accurately detect early changes of macrovascular complications.^33^

Abnormal BMI in this study was associated with macrovascular complications. Obesity among DM patients, including those with T1DM, increases the risk of long-term DM complications, especially ASCVD.^34^ Being underweight has also been reported to be associated with an increased risk of mortality from cardiovascular diseases among adults with DM.^35^ Weight loss in diabetes could indicate possible insulin deficiency, which may lead to suboptimal glycaemic control, increasing the risk of developing DM complications.

In this study higher triglyceride and lower HDL cholesterol levels were associated with macrovascular complications. This pattern of dyslipidaemia linked with qualitative abnormalities of LDL cholesterol is prevalent among young DM patients, more atherogenic, and associated with poor glycaemic control.^36^–^38^ Indeed, the triglyceride-glucose index, a surrogate marker for insulin resistance, is associated with arterial stiffness, an indicator of macrovascular disease.^39^

Hypertension tends to be more common in young individuals with diabetes, particularly T2DM, compared to those without DM.^40^ In this study, although high blood pressure was common, it was not associated with the development of macrovascular complications. The lack of association between some of the established risk factors and diabetes-related complications may be due to the short duration (less than five years) of the diagnosis of diabetes in this study. The onset of DM in young individuals is associated with an increased risk of morbidity and mortality from DM-related vascular complications since the risk factors for these complications usually develop and progress during childhood and adolescence.^6^ Therefore, the importance of a well-structured diabetes education to enhance appropriate self-management and avoid or at least reduce adverse outcomes cannot be overemphasized. A longitudinal follow-up study among children and adolescents with diabetes is required to further explore the burden of chronic diabetes complications among this population. This will provide more information that can be utilized to facilitate the delivery of improved diabetes care in the country and the sub-region.

The inability to perform albuminuria analysis may have underestimated the prevalence of nephropathy in this study. As this was a hospital-based study with few participants, our findings may not be generalizable to the whole population.

## Conclusion

This study shows sub-optimal glycaemic control and a high prevalence of macrovascular (27.6%) and non-ocular microvascular (8.6%) complications among children and adolescents with diabetes in southern Ghana. Long duration of diabetes diagnosis and lower triglyceride levels were associated with non-ocular microvascular complications. In contrast, high triglycerides, lower HDL cholesterol, and abnormal BMI were found to be associated with macrovascular complications. These findings highlight the importance of the early institution of regular screening for diabetes-related complications and their risk factors to allow early detection and appropriate management. There is also the need for healthcare personnel, supported by healthcare administrators at all levels, to intensify their efforts at improving diabetes care among young individuals with diabetes.
